# Prognostic value of neutrophil percentage-to-albumin, uric acid-to-creatinine, and CRP-to-albumin ratios for mortality in patients on maintenance hemodialysis

**DOI:** 10.1590/2175-8239-JBN-2026-0011en

**Published:** 2026-08-24

**Authors:** Tahsin Karaaslan, Ozgul Akdeniz Balseven, Irem Pembegul

**Affiliations:** 1 Goztepe Prof. Dr. Suleyman Yalcin City Hospital, Department of Nephrology, Istanbul, Türkiye.; 2 Malatya Training and Research Hospital, Peritoneal Dialysis and Hemodialysis Unit, Malatya, Türkiye.; 3 Turgut Ozal University, Medical School, Malatya Training and Research Hospital, Department of Nephrology, Malatya, Türkiye.

**Keywords:** Renal Dialysis, Mortality, Uric Acid, C-Reactive Protein, L-Lactate Dehydrogenase

## Abstract

**Objective:**

Identifying reliable biomarkers to predict mortality in hemodialysis patients is essential for risk stratification and clinical decision-making. This study evaluated the prognostic value of the serum uric acid-to-creatinine ratio (SUA/SCr), C-reactive protein-to-albumin ratio (CAR), neutrophil percentage-to-albumin ratio (NPAR), and lactate dehydrogenase (LDH).

**Methods:**

This retrospective cohort study included 138 maintenance hemodialysis patients with at least 6 months of dialysis vintage (2017–2025). Demographic, biochemical, and hematological data were obtained from medical records. Dialysis adequacy was assessed using Kt/Vurea. Mortality predictors were analyzed using ROC curves, logistic regression, and survival analysis.

**Results:**

The mean age of the study population was 60.6 ± 15.1 years, and 50.7% were female. Deceased patients were significantly older than survivors (65.1 ± 16.2 vs. 58.9 ± 14.5 years; p = 0.032). The median hemodialysis vintage was 63.5 months, and 55.1% of patients used a hemodialysis catheter. Mortality was significantly higher among catheter users (p = 0.002). Deceased patients exhibited higher levels of CRP, SUA/SCr, CAR, and NPAR, along with lower albumin levels (all p < 0.001), as well as higher ferritin levels (p = 0.043). ROC analysis identified LDH ≥ 165.5, CAR ≥ 3.1, and NPAR ≥ 18.15 as optimal cut-off values associated with increased mortality risk. In multivariable analysis, CAR, NPAR, LDH, continuous SUA/SCr, and vascular access type were independent predictors of mortality. Kaplan–Meier analysis demonstrated significantly reduced survival in high-risk groups.

**Conclusion:**

Elevated SUA/SCr, CAR, NPAR, and LDH levels are significantly associated with mortality in hemodialysis patients. CAR, NPAR, continuous SUA/SCr, LDH, and catheter use independently predict all-cause mortality.

## INTRODUCTION

Chronic kidney disease (CKD) is characterized by a persistent decline in the estimated glomerular filtration rate below 60 mL/min/1.73 m^2^ for at least three months or by evidence of kidney damage, such as proteinuria or hematuria^1^. In advanced stages, renal replacement therapy becomes necessary when metabolic, volume-related, or uremic complications cannot be adequately controlled^2^. Hemodialysis remains the most frequently utilized renal replacement modality worldwide. Global epidemiological data indicate that approximately 850 million individuals are affected by CKD, and mortality rates among patients undergoing hemodialysis substantially exceed those of the general population^3,4^.

Cardiovascular disease represents the leading cause of death in hemodialysis patients, driven by factors such as hypertension, atherosclerosis, left ventricular hypertrophy, and intradialytic hemodynamic instability^5^. Additionally, vascular access–related infections and chronic inflammatory processes impair immune defense mechanisms, thereby increasing susceptibility to severe infections^6^. Malnutrition, oxidative stress, and protein–energy wasting further exacerbate immune dysfunction and negatively influence clinical outcomes^7^.

Recently, inflammatory and metabolic ratios derived from routine laboratory parameters have gained attention as potential prognostic markers in end-stage renal disease. The serum uric acid-to-creatinine ratio (SUA/SCr) has been linked to oxidative stress and endothelial dysfunction, while the neutrophil percentage-to-albumin ratio (NPAR) reflects both systemic inflammation and nutritional status^8^. Similarly, the C-reactive protein-to-albumin ratio (CAR) integrates inflammatory burden with nutritional impairment and has been associated with adverse outcomes in hemodialysis populations^9,10^.

This study hypothesized that SUA/SCr, CAR, and NPAR are significantly associated with all-cause mortality in patients receiving maintenance hemodialysis. Accordingly, we aimed to investigate the prognostic utility of these indices for predicting mortality in a hemodialysis cohort.

## METHODS

### Study design and participant selection

This study was designed as a retrospective cohort analysis and was conducted at a single tertiary care facility, encompassing the period from January 1, 2017, to June 30, 2025. The cohort comprised clinically stable adult outpatients (aged ≥ 18 years) with end-stage renal disease (ESRD) undergoing a standard maintenance hemodialysis (HD) regimen three times weekly in a dialysis unit setting. All patients requiring catheter-based vascular access utilized permanent (tunneled) catheters. By including only patients with at least six months of dialysis vintage, we aimed to evaluate biomarkers in a stable ambulatory population rather than in acute inpatient settings. Exclusion criteria were applied to patients transferred to external centers without follow-up data, those with incomplete medical records, or individuals receiving immunosuppressive therapies. Furthermore, patients with a known history of solid or hematologic malignancies were excluded from the dataset. This retrospective cohort study was approved by the Malatya Training and Research Hospital Ethics Committee (Approval date: November 6, 2025; Approval No.: E-30785963-020-345392). Due to the retrospective nature of the study, the requirement for informed consent was waived. The study was conducted in accordance with the principles of the Declaration of Helsinki.

### Data acquisition

Data collection was performed through a detailed examination of digital health records and physical patient charts, supplemented by anamnesis directly from patients or their first-degree relatives when clarification was necessary. We recorded essential demographic and clinical parameters, including age, sex, primary etiology of CKD, vascular access type (arteriovenous fistula or permanent [tunneled] catheter), and cumulative duration of hemodialysis therapy. Nutritional status was evaluated using objective clinical markers, including body mass index (BMI) and serum albumin levels. The BMI for each subject was derived by calculating the ratio of weight (kg) to the square of height (m). Regarding laboratory parameters, data for surviving patients were sourced from their latest scheduled monthly check-ups. Conversely, for deceased subjects, laboratory values were retrieved from the final blood samples obtained prior to death. Biochemical and hematological profiles were assessed accordingly. Additionally, the presence of comorbidities— specifically diabetes mellitus, hypertension, coronary artery disease, congestive heart failure, and other pertinent chronic conditions—was recorded.

Complete blood count (CBC) parameters were documented from the most recent monthly assessment for all subjects, encompassing leukocyte and platelet (PLT) counts, hemoglobin (Hb) concentrations, and both absolute counts and percentages for neutrophils, lymphocytes, and monocytes. The Chronic Kidney Disease Epidemiology Collaboration (CKD-EPI) formula was employed to estimate the glomerular filtration rate (eGFR)^11^. The prognostic indices analyzed in this study were derived using the following equations:

Serum uric acid-to-serum creatinine ratio (SUA/SCr) = serum uric acid (mg/dL) / serum creatinine (mg/dL)^12^.

Neutrophil percentage-to-albumin ratio (NPAR) = neutrophil percentage (%) / serum albumin (g/dL)^13^.

C-reactive protein-to-albumin ratio (CAR) = C-reactive protein (mg/L) / serum albumin (g/dL)^14^.

### Assessment of dialysis adequacy

Evaluation of dialysis adequacy was primarily based on kinetic parameter analysis. To achieve this, the Daugirdas equation was utilized to compute Kt/Vurea using blood urea nitrogen (BUN) concentrations from samples drawn pre- and post-hemodialysis. An spKt/V threshold of ≥ 1.2 was regarded as the benchmark for adequate dialysis. Within this formula, K denotes the urea clearance of the dialyzer (mL/min), t signifies the duration of the dialysis session, and V represents the urea distribution volume. The calculation for spKt/V using the Daugirdas method is expressed as:

_sp_Kt/V = -ln[(BUN_post_ / BUN_pre_) - (0.008 × t)] + [(4 - 3.5 × (BUN_post_ / BUN_pre_)) × (UF volume / body weight)]^15^.

### Statistical analysis

Data analysis was performed using Jamovi software. The normality of continuous variables was assessed using the Shapiro–Wilk test. Continuous variables with normal distribution were expressed as mean ± standard deviation (SD), whereas non-normally distributed variables were presented as medians (interquartile range, IQR). Comparisons between two groups were conducted using the Student’s t-test for normally distributed variables and the Mann–Whitney U test for non-normally distributed variables. Categorical variables were expressed as frequencies and percentages and analyzed using the chi-square test. Correlations between continuous variables were assessed using Pearson or Spearman correlation coefficients, as appropriate.

To evaluate mortality, receiver operating characteristic (ROC) curve analysis was performed, and optimal cut-off values were determined by maximizing Youden’s J index. Patients were stratified accordingly. To identify potential risk factors, binary logistic regression analysis was performed, and the results were reported as odds ratios (ORs) with 95% confidence intervals (CIs). Continuous variables were additionally evaluated in categorical form using ROC-derived cut-off values; however, the continuous and categorical forms of the same variable were not included simultaneously in the multivariable model to avoid multicollinearity. Model fit was assessed using the Hosmer–Lemeshow goodness-of-fit test. Variables with a p-value < 0.25 in univariate analysis were subsequently included in the multivariable model.

In addition to logistic regression analyses, time-to-event data were evaluated using Kaplan–Meier survival analysis and compared with the log-rank test.

A post-hoc power analysis was conducted using G*Power software (log-rank test, two-sided, α = 0.05). Based on the observed effect sizes, the minimum sample sizes required to achieve 80% power were approximately 95–136 for SUA/SCr, 65–95 for NPAR, and 55–75 for CAR. Since the study included 138 patients, the sample size was considered adequate to detect significant associations with all-cause mortality. Statistical significance was set at p < 0.05.

## RESULTS

### Demographic and clinical characteristics

The study cohort comprised 138 individuals diagnosed with ESRD who were undergoing thrice-weekly hemodialysis. Women constituted 50.7% (n = 70) of the total cohort. The overall mean age of the cohort was 60.6 ± 15.1 years. Upon analyzing data based on mortality status, deceased subjects were found to be significantly older compared to the surviving group (65.1 ± 16.2 years vs. 58.9 ± 14.5 years; p = 0.032). Regarding body mass index (BMI), the mean value was 23.2 ± 2.9 kg/m^2^, with no statistically significant difference observed between surviving and non-surviving groups (p = 0.383). However, a sex-based comparison revealed that female participants had a significantly higher mean BMI relative to males (24.2 vs. 22.2; p < 0.001). Regarding the primary etiology of chronic kidney disease, diabetes mellitus was the leading cause (32.1%), followed by hypertension (30.2%), glomerulonephritis (14.0%), and polycystic kidney disease (8.1%) (Table 1).

**Table 1:** Demographic characteristics, duration of hemodialysis, and comorbidities according to survival status.

Variable		Total (n = 138)	Survivors (n = 100)	Non-survivors (n = 38)	Test statistic	p value
Age (years)		60.6 ± 15.1	58.9 ± 14.5	65.1 ± 16.2	-2.17^*^	**0.032**
BMI (kg/m^2^)		23.2 ± 2.9	23.0 ± 2.7	23.6 ± 3.4	-0.88^*^	0.383
HD vintage (months)		63.5 (42.0–86.0)	64.5 (40.3–89.3)	62.5 (44.0–78.0)	1826.5^¥^	0.726
Systolic BP (mmHg)		130.0 (100–145)	130.0 (110–145)	110.0 (100–145)	1505.5^¥^	0.059
Diastolic BP (mmHg)		80.0 (60.0–80.0)	80.0 (70.0–80.0)	70.0 (60.0–81.3)	1644.5^¥^	0.212
Sex n (%)	Male	68 (49.3)	52 (76.5)	16 (23.5)	1.08^¶^	0.299
	Female	70 (50.7)	48 (68.6)	22 (31.4)		
Vascular access n (%)	AVF	62 (44.9)	53 (85.5)	9 (14.5)	9.56^¶^	**0.002**
	T.Catheter	76 (55.1)	47 (61.8)	29 (38.2)		
Hypertension n (%)	Present	67 (48.6)	47 (70.1)	20 (29.9)	0.35^¶^	0.554
	Absent	71 (51.4)	53 (74.6)	18 (25.4)		
CAD n (%)	Present	14 (10.1)	9 (64.3)	5 (35.7)	0.52^¶^	0.470
	Absent	124 (89.9)	91 (73.4)	33 (26.6)		
HF n (%)	Present	31 (22.5)	18 (58.1)	13 (41.9)	4.15^¶^	**0.042**
	Absent	107 (77.5)	82 (76.6)	25 (23.4)		
Diabetes n (%)	Present	56 (40.6)	41 (73.2)	15 (26.8)	0.03^¶^	0.870
	Absent	82 (59.4)	59 (72.0)	23 (28.0)		
Primary etiology of CKD n (%)	Diabetes	44 (32.1)	31 (70.5)	13 (29.5)		
	HT	42 (30.2)	31 (73.8)	11 (26.2)		
	GN	19 (14.0)	14 (73.7)	5 (26.3)		
	ADPKD	11 (8.1)	9 (81.8)	2 (18.2)		
	Unknown	22 (15.6)	15 (68.2)	7 (31.8)		

Abbreviations – BMI: body mass index; HD: hemodialysis; BP: blood pressure; CAD: coronary artery disease; HF: heart failure; GN: glomerulonephritis; HT: hypertension; ADPKD: autosomal dominant polycystic kidney disease; T. Catheter: tunneled catheter.

Notes – ¥: Mann–Whitney U test (U);

*: Student’s t-test (t);

¶: chi-square test (^χ2^).

Percentages are calculated by row.

### Hemodynamic and dialysis parameters

Median values for systolic and diastolic blood pressure were 130.0 mmHg (IQR: 100.0–145.0) and 80.0 mmHg (IQR: 70.0–80.0), respectively. No significant differences in either systolic or diastolic blood pressure were observed between the non-surviving and surviving groups (p > 0.05). The median duration of hemodialysis treatment was 63.5 months (IQR: 42.0–86.0), showing no significant difference based on mortality outcomes (p = 0.726). In terms of vascular access, permanent (tunneled) catheters were used by 55.1% (n = 76) of the patients. Regardless of survival status, individuals with an arteriovenous fistula (AVF) exhibited a significantly longer hemodialysis vintage compared to the permanent (tunneled) catheter group (p < 0.01).

### Comorbidities, biochemical and hematological analysis

Detailed biochemical and hematological profiles are presented in Supplementary Table S1. Mortality was significantly associated with lower serum creatinine levels, higher estimated glomerular filtration rate (eGFR), elevated leukocyte counts, absolute neutrophil counts, neutrophil percentages, and lactate dehydrogenase (LDH), along with lower Kt/Vurea values (p < 0.05 for all). While the prevalence of hypertension, coronary artery disease, and diabetes mellitus did not differ significantly between survivors and non-survivors (p > 0.05), mortality rates were significantly higher in patients diagnosed with congestive heart failure (p = 0.042) (Table 1). Compared with survivors, non-survivors had significantly higher CRP, CAR, SUA/SCr, and NPAR levels and lower serum albumin levels (all p < 0.001), as well as higher ferritin levels (p = 0.043) (Table 2).

**Table 2:** Association of acute-phase reactants and inflammatory indices with mortality.

Parameter	Survival status	n	Mean ± SD	Median	IQR	Test statistic	p value
CRP (mg/L)	Survivors	100		6.0	3.0–13.8	858.0^*^	<0.001
	Non-survivors	38		24.2	10.4–92.1		
Ferritin (ng/mL)	Survivors	100		270.0	137.5–601.0	1476.5^*^	0.043
	Non-survivors	38		500.1	177.5–820.8		
Albumin (g/dL)	Survivors	100	3.52 ± 0.5			4.78^†^	<0.001
	Non-survivors	38	2.89 ± 0.8				
CAR	Survivors	100		1.74	0.85–4.19	848.0^*^	<0.001
	Non-survivors	38		8.32	3.31–38.45		
SUA/SCr	Survivors	100		0.66	0.55–0.77	940.0^*^	<0.001
	Non-survivors	38		0.80	0.70–1.00		
NPAR	Survivors	100		17.31	15.6–20.0	1009.5^*^	<0.001
	Non-survivors	38		21.41	18.49–33.22		
Parameter (categorical)	Cut-off value	n	Mortality (%)			χ^2^	p value
CAR	<3.1	8	10.4			25.67^¶^	<0.001
	≥3.1	30	49.2				
SUA/SCr	<0.716	10	13.9			14.05^¶^	<0.001
	≥0.716	28	42.4				
NPAR	<18.15	8	11.6			17.58^¶^	<0.001
	≥18.15	30	43.5				

Abbreviations – n: In the upper section, indicates the total number of patients in each survival group. In the categorical section, n indicates the number of non-survivors within each cut-off category, and mortality (%) represents the proportion of deaths among the total patients in that same category; CRP: C-reactive protein; CAR: C-reactive protein-to-albumin ratio; SUA/SCr: serum uric acid-to-serum creatinine ratio; NPAR: neutrophil percentage-to-albumin ratio; SD: standard deviation; IQR: interquartile range;

¶: chi-square test (^χ2^);

†: Student’s t-test (t);

*: Mann–Whitney U test.

### Predictive analysis and mortality risk factors

**Figure 1:**
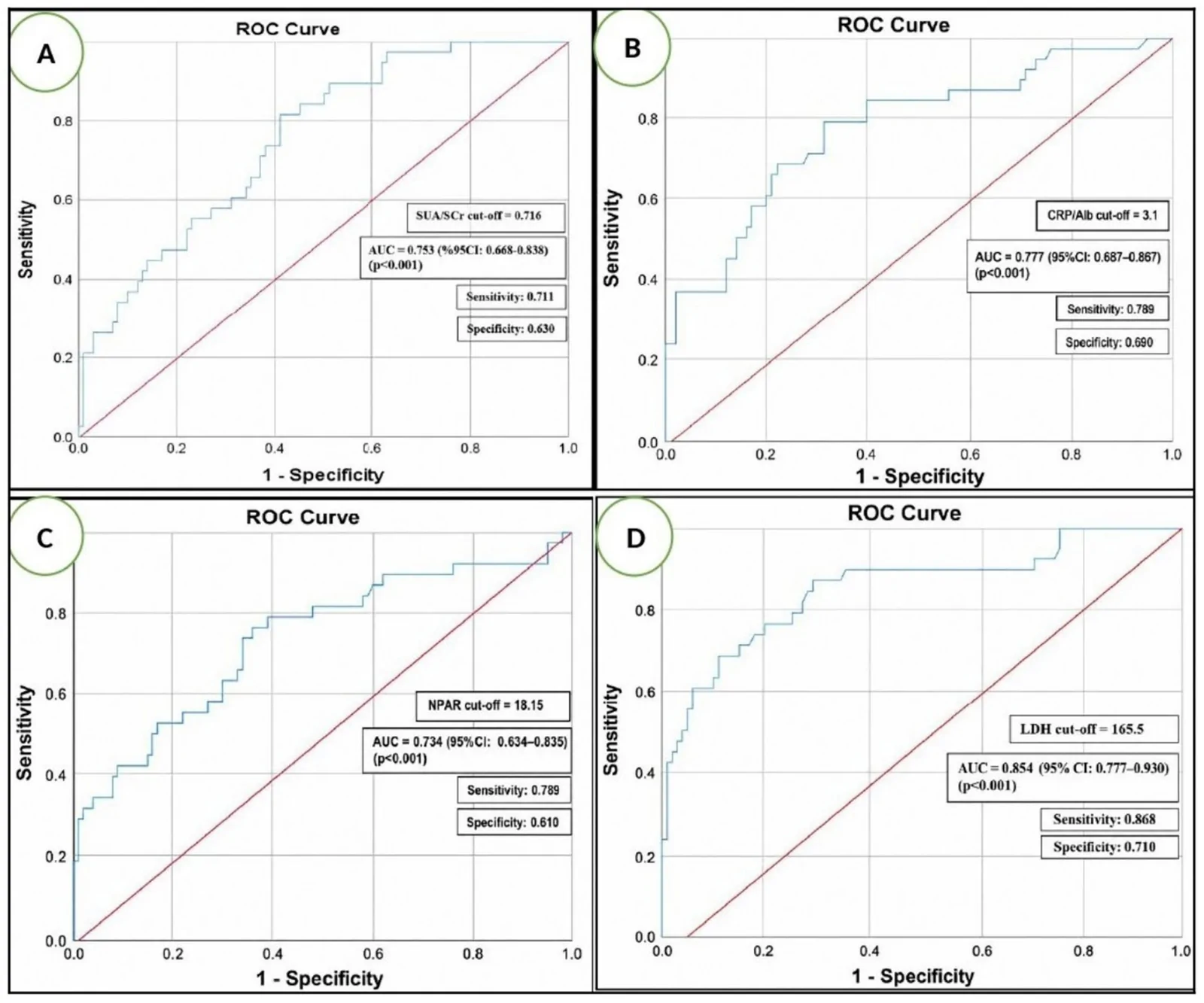
Determination of optimal cut-off values associated with mortality using receiver operating characteristic (ROC) curve analysis.

To ascertain the predictive thresholds for mortality, ROC curve analysis was applied to SUA/SCr, CAR, NPAR, and LDH (Figure 1). Following stratification based on these optimal cut-off points, the data indicated significantly higher mortality rates among patients with CAR ≥ 3.1, SUA/SCr ≥ 0.716, and NPAR ≥ 18.15 (p < 0.001) (Table 2).

Abbreviations – A: Cut-off value of the serum uric acid-to-creatinine ratio (SUA/SCr); B: Cut-off value of the C-reactive protein-to-albumin ratio (CAR); C: Optimal cut-off value of the neutrophil percentage-to-albumin ratio (NPAR); D: Cut-off value of the lactate dehydrogenase (LDH).

Univariate binary logistic regression analysis showed that CRP, SUA/SCr, CAR, and NPAR were significantly associated with increased mortality risk. Ferritin showed a borderline association with mortality (p = 0.050). Specifically, this analysis demonstrated a 4.6-fold increase in mortality risk for SUA/SCr ≥ 0.716, an 8.3-fold increase for CAR ≥ 3.1, and a 5.9-fold increase for NPAR ≥ 18.15 (Table 3).

**Table 3:** Association between each variable and mortality based on univariate binary logistic regression analysis.

Variable	B	S.E.	Wald	df	p value	Exp(B)	95% CI for Exp(B)
Age (years)	0.029	0.014	4.466	1	**0.035**	1.029	1.002–1.057
HF	0.862	0.430	4.027	1	**0.045**	2.369	1.020–5.500
Systolic BP	-0.018	0.009	3.934	1	**0.047**	0.982	0.965–1.000
Diastolic BP	-0.021	0.015	1.955	1	0.162	0.979	0.951–1.008
HbA1c (%)	-0.226	0.159	2.004	1	0.157	0.798	0.584–1.091
Serum creatinine	-0.419	0.098	18.381	1	**<0.001**	0.658	0.543–0.796
eGFR	0.088	0.044	4.025	1	**0.045**	1.092	1.002–1.190
Sodium (mmol/L)	0.084	0.063	1.745	1	0.187	1.087	0.960–1.231
LDH (U/L)	0.012	0.002	25.226	1	**<0.001**	1.012	1.007–1.016
Albumin	-1.744	0.401	18.919	1	**<0.001**	0.175	0.080–0.384
WBC	0.000	0.000	5.896	1	**0.015**	1.000	1.000–1.000
NEU%	0.057	0.019	8.916	1	**0.003**	1.059	1.020–1.100
LYM	0.000	0.000	2.202	1	0.138	1.000	0.999–1.000
CRP (mg/L)	0.029	0.007	14.863	1	**<0.001**	1.029	1.014–1.044
Ferritin	0.001	0.000	3.850	1	**0.050**	1.001	1.000–1.001
Vascular access (catheter)	1.290	0.431	8.962	1	**0.003**	3.634	1.561–8.456
Kt/Vurea	-3.891	1.149	11.466	1	**0.001**	0.020	0.002–0.194
SUA/SCr	2.845	0.914	9.688	1	**0.002**	17.204	2.868–103.198
SUA/SCr (categorical) ≥0.716	1.519	0.422	12.954	1	**<0.001**	4.568	1.997–10.448
CAR	0.072	0.021	11.829	1	**0.001**	1.074	1.031–1.119
CAR (categorical) ≥3.1	2.122	0.453	21.954	1	**<0.001**	8.347	3.436–20.277
NPAR	0.137	0.037	13.861	1	**<0.001**	1.147	1.067–1.233
NPAR (categorical) ≥18.15	1.769	0.448	15.619	1	**<0.001**	5.865	2.439–14.103

Abbreviations – HF: heart failure; BP: blood pressure; WBC: white blood cell count; NEU%: neutrophil percentage; LYM: lymphocyte count; CRP: C-reactive protein; eGFR: estimated glomerular filtration rate; LDH: lactate dehydrogenase; CAR: C-reactive protein-to-albumin ratio; SUA/SCr: serum uric acid-to-creatinine ratio; NPAR: neutrophil percentage-to-albumin ratio. Reference categories were the absence of heart failure, arteriovenous fistula (AVF) for vascular access, and values below the specified cut-off points for categorical variables.

Potential mortality risk factors yielding a p-value < 0.25 in univariate analysis were subsequently incorporated into a multivariable logistic regression model. The final model exhibited a good fit, evidenced by a Hosmer–Lemeshow test result of χ^2^ = 1.67 (p = 0.976). Within this multivariable framework, independent predictors of mortality were identified as categorical LDH ≥ 165.5 (OR = 21.81; p < 0.001), vascular access type (permanent [tunneled] catheter) (OR = 12.17; p = 0.007), serum creatinine level (OR = 0.66; p = 0.013), the SUA/SCr ratio (OR = 4.84; p = 0.013), categorical CAR ≥ 3.1 (OR = 8.62; p = 0.005), and categorical NPAR ≥ 18.15 (OR = 3.81; p = 0.037) (Table 4).

**Table 4:** Association between variables and mortality based on multivariable binary logistic regression analysis.

Variable	B	S.E.	Wald	df	p value	Exp(B)	95% CI for Exp(B)
Age (years)	0.036	0.027	1.77	1	0.183	1.036	0.983–1.092
HF	-0.658	0.798	0.68	1	0.410	0.518	0.108–2.475
Systolic BP	-0.046	0.028	2.72	1	**0.099**	0.955	0.904–1.009
Diastolic BP	0.068	0.050	1.89	1	0.169	1.071	0.971–1.180
HbA1c (%)	-0.394	0.349	1.28	1	0.258	0.674	0.340–1.336
Sodium (mmol/L)	0.153	0.117	1.71	1	0.191	1.165	0.927–1.465
LDH (categorical) ≥165.5	3.082	0.684	20.28	1	**<0.001**	21.808	5.702–83.417
Lymphocyte count	0.000	0.001	0.54	1	0.462	0.998	0.997–1.000
Ferritin	-0.001	0.001	0.63	1	0.429	0.999	0.998–1.001
Vascular access (catheter)	2.499	0.921	7.35	1	**0.007**	12.17	1.999–74.041
Serum creatinine	-0.412	0.166	6.19	1	**0.013**	0.662	0.479–0.916
Kt/Vurea	-1.867	1.946	0.92	1	0.337	0.155	0.003–7.002
WBC	0.000	0.000	3.20	1	0.074	1.000	1.000–1.001
SUA/SCr (continuous)	1.578	0.636	6.15	1	**0.013**	4.843	1.392–16.853
CAR (categorical) ≥3.1	2.154	0.766	7.90	1	**0.005**	8.618	1.920–38.688
NPAR (categorical) ≥18.15	1.336	0.639	4.37	1	**0.037**	3.806	1.087–13.325

Abbreviations – HF: heart Failure; LDH: lactate dehydrogenase; WBC: white blood cell count; SUA/SCr: serum uric acid-to-creatinine ratio; CAR: C-reactive protein-to-albumin ratio; NPAR: neutrophil percentage-to-albumin ratio; BP: blood pressure.

### Survival analysis

**Figure 2:**
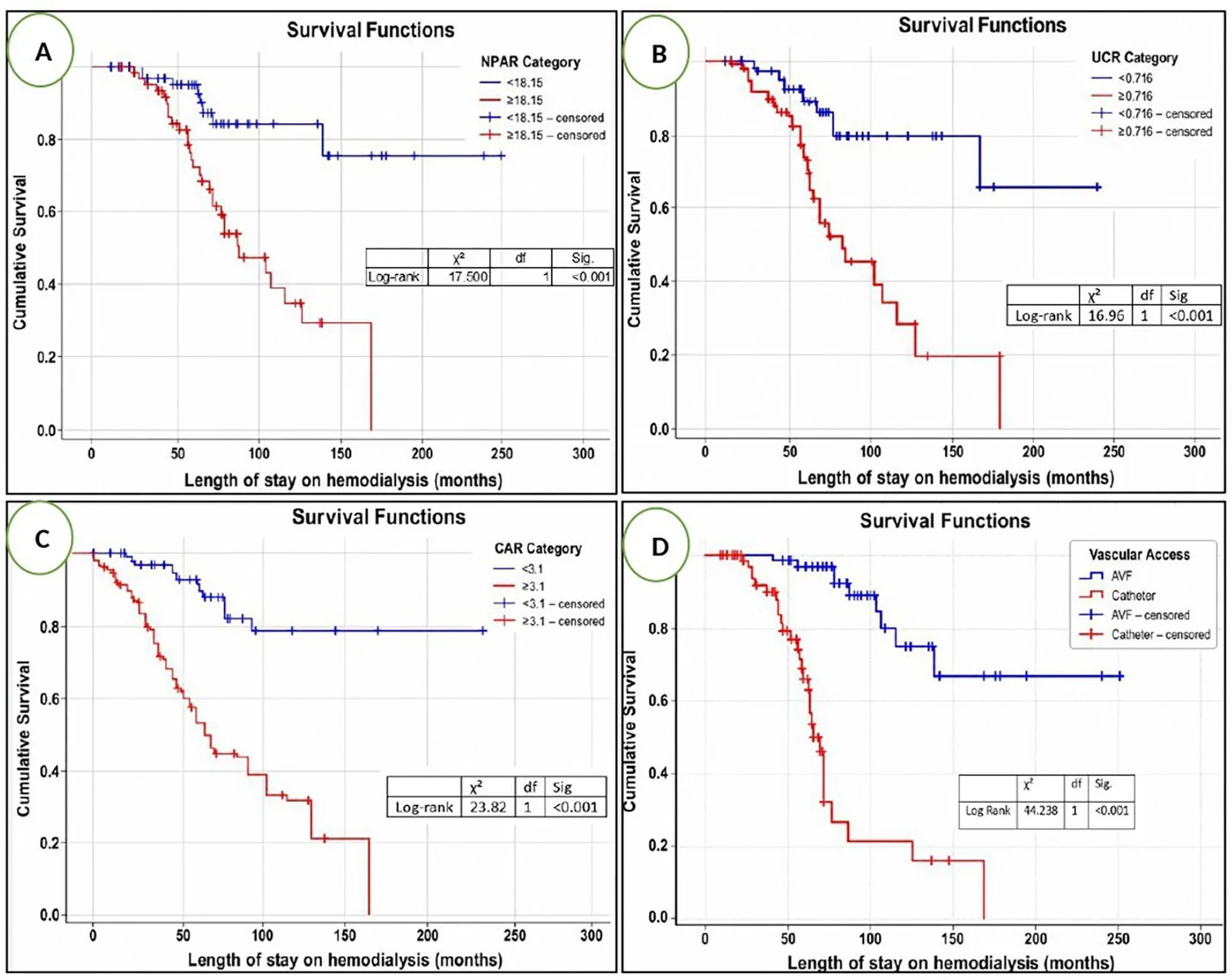
Kaplan–Meier survival analysis of mortality according to inflammatory ratios and vascular access type.

Kaplan–Meier survival analysis revealed statistically significant differences in survival times across patient subgroups defined by the cut-off values for CAR (3.1), SUA/SCr (0.716), and NPAR (18.15), as well as by vascular access type (SUA/SCr: log-rank χ^2^ = 16.96, p < 0.001; CAR: log-rank χ^2^ = 23.82, p < 0.001; NPAR: log-rank χ^2^ = 17.5, p < 0.001; vascular access: log-rank χ^2^ = 44.2, p < 0.001). The corresponding survival curves illustrating these outcomes are depicted in Figure 2.

Abbreviations – A: Kaplan–Meier survival curve according to the categorical values of the neutrophil percentage-to-albumin ratio (NPAR); B: Kaplan–Meier survival curve according to the categorical values of the serum uric acid-to-creatinine ratio (SUA/SCr); C: Kaplan–Meier survival curve according to the categorical values of the C-reactive protein-to-albumin ratio (CAR); D: Kaplan–Meier survival curve according to vascular access type.

## DISCUSSION

This research focused on assessing individuals with end-stage renal disease undergoing a thrice-weekly hemodialysis regimen. Our analysis demonstrated that the non-surviving patient cohort exhibited significantly elevated absolute levels of specific biomarkers, namely the serum uric acid-to-serum creatinine ratio (SUA/SCr), the C-reactive protein-to-albumin ratio (CAR), and the neutrophil percentage-to-albumin ratio (NPAR). Moreover, following the application of ROC-derived cut-off values, multivariable logistic regression models identified CAR and NPAR as independent prognostic factors for mortality.

The results of this study are consistent with the malnutrition– inflammation–atherosclerosis (MIA) syndrome, a well-established concept in prevalent dialysis populations, in which systemic inflammation and nutritional status (reflected by BMI and albumin) act as critical determinants of clinical outcomes. Integrated markers such as CAR and NPAR effectively capture this synergy and serve as independent predictors of mortality risk.

Existing literature indicates that elevated SUA/SCr ratios in patients on chronic hemodialysis are associated with an elevated risk of cardiovascular death^16^. For instance, research by Zhihui Ding et al.^17^ highlighted a robust association between the serum uric acid-to-creatinine ratio and all-cause mortality in geriatric hemodialysis populations, noting its superior predictive capacity compared to serum uric acid or creatinine alone. Consistent with these observations, our data revealed markedly higher SUA/SCr ratios among deceased subjects. While multivariable logistic regression analysis classified the SUA/SCr ratio as an independent mortality predictor (OR = 4.8; p = 0.013), the categorical assessment utilizing the ROC-established threshold of 0.716 did not yield a statistically significant association with mortality in the multivariable model (p = 0.380). Dichotomization of continuous variables may lead to a loss of prognostic information, which may explain the discrepancy observed for SUA/SCr.

Research has suggested that elevated CAR levels are significantly associated with both cardiovascular and allcause mortality, supporting CAR as a viable independent prognostic marker for patients with stage 3–5 chronic kidney disease^18^. Additional studies have characterized serum CAR as a composite measure reflecting both nutritional status and systemic inflammatory load, linking it to elevated mortality risk in hemodialysis cohorts^10^. Our findings are consistent with these reports, showing significantly elevated CAR values in the non-surviving group (p < 0.001). Through multivariable logistic regression, CAR emerged as an independent predictor of death. Specifically, when analyzing data categorically using the ROC-determined cut-off of 3.1, individuals with CAR ≥ 3.1 had 8.6-fold higher odds of mortality (OR = 8.6; p = 0.005). Furthermore, Kaplan–Meier survival curves confirmed substantially reduced overall survival among patients above this CAR threshold (log-rank χ^2^ = 23.82; p < 0.001).

Investigations by Changjing Shi et al.^19^ demonstrated that patients presenting with a neutrophil percentage-to-albumin ratio (NPAR) above a cut-off of 16.96 experienced markedly inferior survival outcomes, identifying NPAR as an independent mortality predictor. In our analysis, subjects with NPAR ≥ 18.15 had 3.8-fold higher odds of mortality (OR = 3.8; p = 0.037), and multivariable modeling confirmed NPAR as an independent predictor. Consistent with this, the Kaplan–Meier survival analysis evidenced significantly reduced survival among patients with NPAR ≥ 18.15 (log-rank χ^2^ = 17.5; p < 0.001).

Naoki Kimata et al.^20^ demonstrated that reductions in Kt/Vurea values are significantly associated with increased mortality, especially in female hemodialysis patients, identifying this reduction as an independent risk factor. Consistent with Kimata et al.’s observations, our study found that Kt/V values, serving as a metric for dialysis adequacy, were significantly lower in the non-surviving cohort relative to survivors (p < 0.001). Nevertheless, our multivariable regression model failed to identify a significant association between Kt/V and mortality; consequently, Kt/V was not classified as an independent predictor of death in this analysis (p = 0.337).

We observed a mortality rate of 38.2% in patients utilizing catheters for hemodialysis, in contrast to 14.5% among those employing an arteriovenous fistula (AVF). Chi-square analysis confirmed this difference to be statistically significant (χ^2^ = 9.56; p = 0.002). Through multivariable binary logistic regression, the vascular access type emerged as an independent risk factor, with catheter usage being associated with 12.2-fold higher odds of mortality (OR = 12.2; p = 0.007). Additionally, Kaplan–Meier analysis revealed that catheter users experienced significantly shorter survival compared to the AVF group (log-rank χ^2^ = 44.2; p < 0.001). These results underscore the critical role of vascular access type in determining the prognosis for hemodialysis patients. Previous research is consistent with these findings, reporting elevated mortality rates associated with catheter-based access relative to arteriovenous fistulas^21,22^.

Elevated levels of lactate dehydrogenase (LDH) have been linked to all-cause mortality in prior investigations^23^. For example, Verena Gotta et al.^24^ noted that increased LDH concentrations in young adult and pediatric chronic hemodialysis patients could serve as a potent mortality marker, reflecting a complex burden of risk. Supporting these findings, our study detected significantly higher LDH levels in non-survivors compared to the surviving group (p < 0.001). Kaplan– Meier survival analysis demonstrated that individuals with LDH ≥ 165.5 exhibited significantly reduced survival (log-rank χ^2^ = 26.04; p < 0.001). Furthermore, those with LDH values ≥ 165.5 had 21.8-fold higher odds of mortality (OR = 21.808; p < 0.001), with multivariable logistic regression confirming LDH as an independent mortality predictor.

### Limitations of the study

This study has several limitations that should be considered when interpreting the findings. First, its retrospective, single-center design limits generalizability and precludes causal inference. Second, the relatively small sample size and limited number of mortality events may increase the risk of overfitting in multivariable models. Third, inflammatory and metabolic biomarkers were assessed at a single time point; therefore, their longitudinal variability could not be evaluated, although serial measurements might have provided more robust prognostic information. Fourth, the retrospective design precluded full adjustment for potential confounders, including medication use (e.g., statins, erythropoiesis-stimulating agents, or uric acid–lowering therapies), residual renal function, and ultrafiltration parameters. In addition, only all-cause mortality was evaluated, preventing analysis of cause-specific outcomes (e.g., cardiovascular or infection-related deaths) and limiting mechanistic interpretation. Finally, survival analyses were restricted to Kaplan–Meier methods without Cox proportional hazards modeling, which may limit assessment of time-dependent effects. Therefore, external validation in large, prospective, multicenter cohorts is warranted.

## CONCLUSION

In conclusion, this study comprehensively evaluated biomarkers associated with mortality in patients undergoing hemodialysis and demonstrated that elevated levels of SUA/SCr, the C-reactive protein (CRP)/albumin ratio (CAR), neutrophil percentage-to-albumin ratio (NPAR), and LDH were significantly associated with increased mortality. Patients with biomarker levels above the ROC-derived cut-off values exhibited markedly reduced survival rates in Kaplan–Meier analyses. Furthermore, in multivariable logistic regression models, continuous SUA/SCr, CAR, NPAR, and LDH were identified as independent predictors of mortality.

In addition, the use of a central venous catheter was associated with a significantly higher mortality risk compared with an arteriovenous fistula. These findings emphasize the value of an integrated prognostic approach in hemodialysis patients that considers inflammation, nutritional status, cellular injury markers, and vascular access type. Readily available and cost-effective biomarkers may aid in risk stratification; however, confirmation in large, prospective, multicenter studies is required.

## Data Availability

The data that support the findings of this study are available on request from the corresponding author.

## SUPPLEMENTARY MATERIAL

The following online material is available for this article:

**Table S1** – Association of laboratory parameters with mortality.
