# Supplementary material for: Prognostic value of neutrophil percentage-to-albumin, uric acid-to-creatinine, and CRP-to-albumin ratios for mortality in patients on maintenance hemodialysis
Source: J Bras Nefrol. 2026 Aug 24;48(4):e20260011. doi: 10.1590/2175-8239-JBN-2026-0011en (PMC13544940; doi:10.1590/2175-8239-JBN-2026-0011en)
Supplement: Supplementary file 1 [file 2175-8239-jbn-48-4-e20260011-s1.pdf]

Braz. J. Nephrol.
https://doi.org/10.1590/2175-8239-JBN-2026-0011en

Supplementary Material to “Prognostic value of neutrophil percentage-to-albumin, uric acid-to-creatinine and CRP-to-albumin ratios for mortality in patients on maintenance hemodialysis”

TABLE S1 Association of laboratory parameters with mortality.

Parameter	**Survivors (n=100)**	**Deceased (n=38)**	**Statistic**	**p**
Glucose (mg/dL)	132.8 ± 83.9	128.7 ± 71.9	t = 0.27	0.787
Creatinine (mg/dL)	9.07 ± 2.8	6.51 ± 2.3	t = 5.01	**<0.001**
eGFR (mL/min/1.73m²)	6.5 ± 4.6	9.4 ± 8.3	t = −2.10	**0.041**
Sodium (mmol/L)	136.3 ± 3.1	137.1 ± 3.3	t = −1.32	0.186
Potassium (mmol/L)	5.1 ± 0.8	5.2 ± 1.1	t = −0.44	0.659
Uric acid (mg/dL)	5.81 ± 1.1	5.72 ± 1.3	t = 0.42	0.673
Phosphate (mg/dL)	5.0 ± 1.3	4.9 ± 1.9	t = 0.07	0.942
Hemoglobin (g/dL)	10.1 ± 1.5	10.1 ± 1.5	t = −0.12	0.902
WBC (×10³/µL)	6.7 ± 2.5	8.8 ± 5.9	t = −2.11	**0.040**
Neutrophils (×10³/µL)	4.3 ± 2.0	5.9 ± 4.0	t = −2.40	**0.021**
Neutrophil (%)	61.6 ± 10.1	68.5 ± 14.6	t = −2.68	**0.010**
Lymphocytes (×10³/µL)	1.7 ± 1.3	1.4 ± 0.8	t = 1.32	0.188
Monocytes (×10³/µL)	0.56 ± 0.25	0.55 ± 0.24	t = 0.19	0.849
Platelets (×10⁹/L)	184.7 ± 65.0	200.1 ± 100.4	t = −1.06	0.292
Kt/V	1.75 ± 0.19	1.62 ± 0.18	t = 3.70	**<0.001**
LDH (U/L)	140 (112–182)	304 (190–510)	U = 556.0	**<0.001**
HbA1c (%)	5.60 (5.1–6.5)	5.65 (4.9–6.3)	U = 1721.0	0.393

Data are presented as mean ± standard deviation (SD) or median (interquartile range, IQR), as appropriate. Comparisons between groups were performed using Student’s t-test or Mann–Whitney U test, as appropriate.

Abbreviations - eGFR, estimated glomerular filtration rate; Kt/V, dialysis adequacy index; LDH, lactate dehydrogenase; HbA1c, glycated hemoglobin; SD, standard deviation; IQR, interquartile range. Tests: t, Student’s t-test; U, Mann–Whitney U test.
