# Supplementary material for: Prognostic value of neutrophil percentage-to-albumin, uric acid-to-creatinine, and CRP-to-albumin ratios for mortality in patients on maintenance hemodialysis
Source: J Bras Nefrol. 2026 Aug 24;48(4):e20260011. doi: 10.1590/2175-8239-JBN-2026-0011en (PMC13544940; doi:10.1590/2175-8239-JBN-2026-0011en)
Supplement: Supplementary file 2 [file 2175-8239-jbn-48-4-e20260011-s2.pdf]

Braz. J. Nephrol.
https://doi.org/10.1590/2175-8239-JBN-2026-0011pt

Material suplementar de “Valor prognóstico das razões percentual de neutrófilos/albumina, ácido úrico/creatinina e PCR/albumina para mortalidade em pacientes em hemodiálise de manutenção”

TABELA S1 Associação dos parâmetros laboratoriais com a mortalidade.

Parâmetro	Sobreviventes (n=100)	Óbitos (n=38)	Estatística	p
**Glicose (mg/dL)**	132,8 ± 83,9	128,7 ± 71,9	t = 0,27	0,787
**Creatinina (mg/dL)**	9,07 ± 2,8	6,51 ± 2,3	t = 5,01	**<0,001**
**TFGe (mL/min/1,73 m²)**	6,5 ± 4,6	9,4 ± 8,3	t = −2,10	**0,041**
**Sódio (mmol/L)**	136,3 ± 3,1	137,1 ± 3,3	t = −1,32	0,186
**Potássio (mmol/L)**	5,1 ± 0,8	5,2 ± 1,1	t = −0,44	0,659
**Ácido úrico (mg/dL)**	5,81 ± 1,1	5,72 ± 1,3	t = 0,42	0,673
**Fosfato (mg/dL)**	5,0 ± 1,3	4,9 ± 1,9	t = 0,07	0,942
**Hemoglobina (g/dL)**	10,1 ± 1,5	10,1 ± 1,5	t = −0,12	0,902
**Leucócitos (×10³/µL)**	6,7 ± 2,5	8,8 ± 5,9	t = −2,11	**0,040**
**Neutrófilos (×10³/µL)**	4,3 ± 2,0	5,9 ± 4,0	t = −2,40	**0,021**
**Neutrófilos (%)**	61,6 ± 10,1	68,5 ± 14,6	t = −2,68	**0,010**
**Linfócitos (×10³/µL)**	1,7 ± 1,3	1,4 ± 0,8	t = 1,32	0,188
**Monócitos (×10³/µL)**	0,56 ± 0,25	0,55 ± 0,24	t = 0,19	0,849
**Plaquetas (×10⁹/L)**	184,7 ± 65,0	200,1 ± 100,4	t = −1,06	0,292
**Kt/V**	1,75 ± 0,19	1,62 ± 0,18	t = 3,70	**<0,001**
**LDH (U/L)**	140 (112–182)	304 (190–510)	U = 556,0	**<0,001**
**HbA1c (%)**	5,60 (5,1–6,5)	5,65 (4,9–6,3)	U = 1721,0	0,393

Os dados são apresentados como média ± desvio padrão (DP) ou mediana (intervalo interquartil, IIQ), conforme apropriado. As comparações entre os grupos foram realizadas usando o teste t de Student ou o teste U de Mann-Whitney, conforme apropriado.

Abreviações - TFGe, taxa de filtração glomerular estimada; Kt/V, índice de adequação da diálise; LDH, lactato desidrogenase; HbA1c, hemoglobina glicada; DP, desvio padrão; IIQ, intervalo interquartil. Testes: t, teste t de Student; U, teste U de Mann-Whitney.
