# Supplementary material for: Prognostic value of neutrophil percentage-to-albumin, uric acid-to-creatinine, and CRP-to-albumin ratios for mortality in patients on maintenance hemodialysis
Source: J Bras Nefrol. 2026 Aug 24;48(4):e20260011. doi: 10.1590/2175-8239-JBN-2026-0011en (PMC13544940; doi:10.1590/2175-8239-JBN-2026-0011en)
Supplement: Translation

# Translation: Valor prognóstico das razões percentual de neutrófilos/albumina, ácido úrico/creatinina, e proteína C reativa/albumina para mortalidade em pacientes em hemodiálise de manutenção

**DOI:** 10.1590/2175-8239-JBN-2026-0011pt

**Authors:** Tahsin Karaaslan, Ozgul Akdeniz Balseven, Irem Pembegul

**Affiliations:** 1 Goztepe Prof. Dr. Suleyman Yalcin City Hospital, Department of Nephrology, Istanbul, Türkiye.; 2 Malatya Training and Research Hospital, Peritoneal Dialysis and Hemodialysis Unit, Malatya, Türkiye.; 3 Turgut Ozal University, Medical School, Malatya Training and Research Hospital, Department of Nephrology, Malatya, Türkiye.

**Keywords:** Diálise Renal, Mortalidade, Ácido Úrico, Proteína C-Reativa, L-Lactato Desidrogenase

## Abstract

**Objetivo:**

A identificação de biomarcadores confiáveis para prever mortalidade em pacientes em hemodiálise é essencial para a estratificação de risco e a tomada de decisão clínica. Este estudo avaliou o valor prognóstico da razão ácido úrico sérico/creatinina (SUA/SCr), da razão proteína C reativa/albumina (CAR), da razão percentual de neutrófilos/albumina (NPAR) e da desidrogenase láctica (LDH).

**Métodos:**

Este estudo de coorte retrospectivo incluiu 138 pacientes em hemodiálise de manutenção há pelo menos 6 meses (2017–2025). Dados demográficos, bioquímicos e hematológicos foram obtidos a partir de prontuários médicos. A adequação da diálise foi avaliada utilizando Kt/Vurea. Os preditores de mortalidade foram analisados por curvas ROC, regressão logística e análise de sobrevida.

**Resultados:**

A idade média da população do estudo foi de 60,6 ± 15,1 anos, e 50,7% eram do sexo feminino. Os pacientes que evoluíram a óbito eram significativamente mais velhos do que os sobreviventes (65,1 ± 16,2 vs. 58,9 ± 14,5 anos; p = 0,032). A mediana da duração da hemodiálise foi de 63,5 meses, e 55,1% dos pacientes utilizavam cateter de hemodiálise. A mortalidade foi significativamente maior entre os usuários de cateter (p = 0,002). Os pacientes falecidos apresentaram níveis mais elevados de PCR, SUA/SCr, CAR e NPAR e níveis mais baixos de albumina (todos com p < 0,001), além de níveis mais elevados de ferritina (p = 0,043). A análise ROC identificou LDH ≥ 165,5, CAR ≥ 3,1 e NPAR ≥ 18,15 como valores de corte ideais associados ao aumento do risco de mortalidade. Na análise multivariada, CAR, NPAR, LDH, SUA/SCr contínuo e o tipo de acesso vascular foram preditores independentes de mortalidade. A análise de *Kaplan–Meier* demonstrou redução significativa da sobrevida nos grupos de alto risco.

**Conclusão:**

Níveis elevados de SUA/SCr, CAR, NPAR e LDH estão significativamente associados à mortalidade em pacientes em hemodiálise. CAR, NPAR, SUA/SCr contínuo, LDH e o uso de cateter predizem de forma independente a mortalidade por todas as causas

## INTRODUÇÃO

A doença renal crônica (DRC) é caracterizada por uma redução persistente da taxa de filtração glomerular estimada abaixo de 60 mL/min/1,73 m^2^ por pelo menos três meses ou pela presença de evidência de dano renal, como proteinúria ou hematúria^1^. Em estágios avançados, a terapia de substituição renal torna-se necessária quando complicações metabólicas, relacionadas ao volume ou urêmicas não podem ser controladas adequadamente^2^. A hemodiálise continua sendo a modalidade de substituição renal mais frequentemente utilizada em todo o mundo. Dados epidemiológicos globais indicam que aproximadamente 850 milhões de indivíduos são afetados pela DRC e que as taxas de mortalidade entre pacientes submetidos à hemodiálise excedem substancialmente aquelas observadas na população geral^3,4^.

As doenças cardiovasculares representam a principal causa de óbito em pacientes em hemodiálise, impulsionadas por fatores como hipertensão, aterosclerose, hipertrofia ventricular esquerda e instabilidade hemodinâmica intradialítica^5^. Além disso, infecções relacionadas ao acesso vascular e processos inflamatórios crônicos comprometem os mecanismos de defesa imunológica, aumentando a suscetibilidade a infecções graves^6^. A desnutrição, o estresse oxidativo e o desgaste proteico-energético agravam ainda mais a disfunção imunológica e influenciam negativamente os desfechos clínicos^7^.

Recentemente, razões inflamatórias e metabólicas derivadas de parâmetros laboratoriais de rotina têm recebido atenção como potenciais marcadores prognósticos na doença renal em estágio terminal. A razão ácido úrico sérico/creatinina (SUA/SCr) tem sido associada ao estresse oxidativo e à disfunção endotelial, enquanto a razão percentual de neutrófilos/albumina (NPAR) reflete tanto a inflamação sistêmica quanto o estado nutricional^8^. De forma semelhante, a razão proteína C reativa/albumina (CAR) integra a carga inflamatória com o comprometimento nutricional e tem sido associada a desfechos adversos em populações em hemodiálise^9,10^.

Este estudo partiu da hipótese de que SUA/SCr, CAR e NPAR estão significativamente associados à mortalidade por todas as causas em pacientes submetidos à hemodiálise de manutenção. Assim, objetivamos investigar a utilidade prognóstica desses índices na predição de mortalidade em uma coorte de pacientes em hemodiálise.

## MÉTODOS

### Desenho do estudo e seleção dos participantes

Este estudo foi delineado como uma análise de coorte retrospectiva e conduzido em um único centro terciário, abrangendo o período de 1º de janeiro de 2017 a 30 de junho de 2025. A coorte foi composta por pacientes ambulatoriais adultos clinicamente estáveis (idade ≥ 18 anos) com doença renal em estágio terminal (DRET), submetidos a um regime padrão de hemodiálise (HD) de manutenção três vezes por semana em uma unidade de diálise. Todos os pacientes que necessitaram de acesso vascular por cateter utilizaram cateteres permanentes (tunelizados). Ao incluir apenas pacientes com pelo menos seis meses em diálise, nosso objetivo foi avaliar biomarcadores em uma população ambulatorial estável, em vez de cenários hospitalares agudos.

Os critérios de exclusão foram aplicados a pacientes transferidos para centros externos sem dados de seguimento, aqueles com registros médicos incompletos ou indivíduos em uso de terapias imunossupressoras. Além disso, pacientes com histórico conhecido de neoplasias sólidas ou hematológicas foram excluídos do conjunto de dados.

Este estudo foi aprovado pelo Comitê de Ética do *Malatya Training and Research Hospital* (Aprovação nº: E-30785963-020-345392; data: 6 de novembro de 2025). Devido ao caráter retrospectivo do estudo, a exigência de consentimento informado foi dispensada. O estudo foi conduzido de acordo com os princípios éticos da Declaração de Helsinque.

### Coleta de dados

A coleta de dados foi realizada por meio de uma análise detalhada de registros eletrônicos de saúde e prontuários físicos dos pacientes, complementada por anamnese diretamente com os pacientes ou seus familiares de primeiro grau, quando necessário. Foram registrados parâmetros demográficos e clínicos essenciais, incluindo idade, sexo, etiologia primária da DRC, tipo de acesso vascular (fístula arteriovenosa ou cateter permanente [tunelizado]) e duração cumulativa da terapia de hemodiálise.

O estado nutricional foi avaliado por meio de marcadores clínicos objetivos, incluindo o índice de massa corporal (IMC) e os níveis séricos de albumina. O IMC de cada indivíduo foi calculado como a razão entre o peso (kg) e o quadrado da altura (m).

Em relação aos dados laboratoriais, para os pacientes sobreviventes foram utilizados os resultados dos exames mensais mais recentes. Para os pacientes falecidos, os valores laboratoriais foram obtidos a partir das últimas amostras de sangue coletadas antes do óbito. Os perfis bioquímicos e hematológicos foram avaliados conforme descrito.

Além disso, a presença de comorbidades — especificamente diabetes mellitus, hipertensão, doença arterial coronariana, insuficiência cardíaca congestiva e outras condições crônicas relevantes — foi registrada.

Os parâmetros do hemograma completo (CBC) foram documentados a partir da avaliação mensal mais recente para todos os pacientes, incluindo contagem de leucócitos, plaquetas (PLT), níveis de hemoglobina (Hb), bem como contagens absolutas e percentuais de neutrófilos, linfócitos e monócitos. A fórmula da *Chronic Kidney Disease Epidemiology Collaboration* (CKD-EPI) foi utilizada para estimar a taxa de filtração glomerular (TFGe)^11^. Os índices prognósticos analisados neste estudo foram calculados por meio das seguintes equações:

Razão ácido úrico sérico/creatinina sérica (SUA/SCr) = ácido úrico sérico (mg/dL) /creatinina sérica (mg/dL)^12^.

Razão porcentagem de neutrófilos/albumina (NPAR) = percentual de neutrófilos (%) /albumina sérica (g/dL)^13^.

Razão proteína C reativa/albumina (CAR) = proteína C reativa (mg/L) /albumina sérica (g/dL)^14^.

### Avaliação da adequação da diálise

A avaliação da adequação da diálise foi baseada principalmente na análise de parâmetros cinéticos. Para isso, foi utilizada a equação de Daugirdas para calcular o Kt/Vureia a partir das concentrações de nitrogênio ureico no sangue (BUN) obtidas antes e após a sessão de hemodiálise. Um valor de spKt/V ≥ 1,2 foi considerado o padrão para uma diálise adequada.

Nesta fórmula, K representa a depuração do dialisador de ureia (mL/min), t corresponde à duração da sessão de diálise e V representa o volume de distribuição da ureia. O cálculo do spKt/V pelo método de Daugirdas é expresso da seguinte forma:

_sp_Kt/V = -ln[(BUN_post_ /BUN_pre_) -(0,008 × t)] + [(4 -3,5 × (BUN_post_ /BUN_pre_)) × (volume de UF /peso corporal)]^15^.

### Análise estatística

A análise dos dados foi realizada utilizando o software Jamovi. A normalidade das variáveis contínuas foi avaliada pelo teste de Shapiro–Wilk. Variáveis contínuas com distribuição normal foram expressas como média ± desvio padrão (DP), enquanto variáveis não normalmente distribuídas foram apresentadas como mediana (intervalo interquartil, IIQ).

As comparações entre dois grupos foram realizadas utilizando o teste t de *Student* para variáveis normalmente distribuídas e o teste de *Mann–Whitney U* para variáveis não normalmente distribuídas. Variáveis categóricas foram expressas como frequências e porcentagens e analisadas pelo teste do qui-quadrado. Correlações entre variáveis contínuas foram avaliadas utilizando os coeficientes de correlação de *Pearson* ou *Spearman*, conforme apropriado.

Para avaliar a mortalidade, foi realizada análise por curvas ROC (*receiver operating characteristic*), e os valores de corte ideais foram determinados maximizando o índice de *Youden* (J). Os pacientes foram estratificados de acordo com esses valores de corte.

Para identificar potenciais fatores de risco, foi realizada análise de regressão logística binária, e os resultados foram apresentados como *odds ratios* (ORs) com intervalos de confiança de 95% (ICs). Variáveis contínuas também foram avaliadas de forma categórica utilizando os valores de corte derivados da análise ROC; no entanto, as formas contínuas e categóricas da mesma variável não foram incluídas simultaneamente no modelo multivariado para evitar multicolinearidade.

O ajuste do modelo foi avaliado pelo teste de *Hosmer–Lemeshow*. Variáveis com valor de p < 0,25 na análise univariada foram posteriormente incluídas no modelo multivariado. Além das análises de regressão logística, dados de tempo até o evento foram avaliados utilizando análise de sobrevida de *Kaplan–Meier* e comparados pelo teste log-rank.

Uma análise de poder pós-hoc foi realizada utilizando o software G*Power (teste *log-rank*, bilateral, α = 0,05). Com base nos tamanhos de efeito observados, os tamanhos mínimos de amostra necessários para atingir 80% de poder estatístico foram aproximadamente 95–136 para SUA/SCr, 65–95 para NPAR e 55–75 para CAR. Como o estudo incluiu 138 pacientes, o tamanho amostral foi considerado adequado para detectar associações significativas com mortalidade por todas as causas. O nível de significância estatística foi estabelecido em p < 0,05.

## RESULTADOS

### Características demográficas e clínicas

A coorte do estudo foi composta por 138 indivíduos com DRET submetidos à hemodiálise três vezes por semana. As mulheres representaram 50,7% (n = 70) da população total. A idade média geral foi de 60,6 ± 15,1 anos. Os pacientes que evoluíram a óbito eram significativamente mais velhos do que os sobreviventes (65,1 ± 16,2 vs. 58,9 ± 14,5 anos; p = 0,032).

O índice de massa corporal (IMC) médio foi de 23,2 ± 2,9 kg/m^2^, sem diferença significativa entre sobreviventes e não sobreviventes (p = 0,383). No entanto, as participantes do sexo feminino apresentaram IMC médio significativamente maior em comparação aos homens (24,2 vs. 22,2; p < 0,001).

Em relação à etiologia primária da doença renal crônica, o diabetes mellitus foi a principal causa (32,1%), seguido por hipertensão (30,2%), glomerulonefrite (14,0%) e doença renal policística (8,1%) (Tabela 1).

**Tabela 1:** Características demográficas, duração da hemodiálise e comorbidades de acordo com o estado de sobrevida.

Variável		Total (n = 138)	Sobreviventes (n = 100)	Não sobreviventes (n = 38)	Estatística do teste	p
Idade (anos)		60,6 ± 15,1	58,9 ± 14,5	65,1 ± 16,2	-2,17^*^	**0,032**
IMC (kg/m^2^)		23,2 ± 2,9	23,0 ± 2,7	23,6 ± 3,4	-0,88^*^	0,383
Duração da HD		63,5	64,5	62,5	1826,5^¥^	0,726
(meses)		(42,0–86,0)	(40,3–89,3)	(44,0–78,0)		
PA sistólica		130,0	130,0	110,0	1505,5^¥^	0,059
(mmHg)		(100–145)	(110–145)	(100–145)		
PA diastólica		80,0	80,0	70,0	1644,5^¥^	0,212
(mmHg)		(60,0–80,0)	(70,0–80,0)	(60,0–81,3)		
Sexo n (%)	M	68 (49,3)	52 (76,5)	16 (23,5)	1,08^¶^	0,299
	F	70 (50,7)	48 (68,6)	22 (31,4)		
Acesso vascular	AVF	62 (44,9)	53 (85,5)	9 (14,5)	9,56^¶^	**0,002**
n (%)						
	T.C.	76 (55,1)	47 (61,8)	29 (38,2)		
Hipertensão n (%)	Presente	67 (48,6)	47 (70,1)	20 (29,9)	0,35^¶^	0,554
	Ausente	71 (51,4)	53 (74,6)	18 (25,4)		
CAD n (%)	Presente	14 (10,1)	9 (64,3)	5 (35,7)	0,52^¶^	0,470
	Ausente	124 (89,9)	91 (73,4)	33 (26,6)		
HF n (%)	Presente	31 (22,5)	18 (58,1)	13 (41,9)	4,15^¶^	**0,042**
	Ausente	107 (77,5)	82 (76,6)	25 (23,4)		
Diabetes n (%)	Presente	56 (40,6)	41 (73,2)	15 (26,8)	0,03^¶^	0,870
	Ausente	82 (59,4)	59 (72,0)	23 (28,0)		
Etiologia primária da DRC	Diabetes	44 (32,1)	31 (70,5)	13 (29,5)		
	HT	42 (30,2)	31 (73,8)	11 (26,2)		
	GN	19 (14,0)	14 (73,7)	5 (26,3)		
	ADPKD	11 (8,1)	9 (81,8)	2 (18,2)		
	Desconh.	22 (15,6)	15 (68,2)	7 (31,8)		

Abreviaturas – HD: hemodiálise; IMC: índice de massa corporal; PA: pressão arterial; AVF: fístula arteriovenosa; T.C.: cateter tunelizado; CAD: doença arterial coronariana; HF: insuficiência cardíaca; DRC: doença renal crônica; GN: glomerulonefrite; ADPKD: doença renal policística autossômica dominante.

Notas – ¥: teste de *Mann–Whitney* (U);

*: teste t de *Student* (t);

¶: teste do qui-quadrado (χ^2^).

As porcentagens são calculadas por linha.

### Parâmetros hemodinâmicos e de diálise

As medianas das pressões arteriais sistólica e diastólica foram de 130,0 mmHg (IIQ: 100,0–145,0) e 80,0 mmHg (IIQ: 70,0–80,0), respectivamente, sem diferenças significativas entre sobreviventes e não sobreviventes (p > 0,05). A duração mediana da hemodiálise foi de 63,5 meses (IIQ: 42,0–86,0), sem diferença significativa entre os grupos (p = 0,726).

Cateteres permanentes (tunelizados) foram utilizados em 55,1% (n = 76) dos pacientes. Pacientes com acesso por fístula arteriovenosa (AVF) apresentaram tempo de diálise significativamente maior em comparação àqueles com cateteres tunelizados (p < 0,01).

### Comorbidades, análise bioquímica e hematológica

Os parâmetros bioquímicos e hematológicos detalhados estão apresentados na Tabela Suplementar S1. A mortalidade esteve significativamente associada a níveis mais baixos de creatinina sérica, valores mais elevados da taxa de filtração glomerular estimada (TFGe), contagens mais elevadas de leucócitos, contagem absoluta de neutrófilos, percentual de neutrófilos e níveis mais elevados de desidrogenase láctica (LDH), bem como a valores mais baixos de Kt/Vureia (todos p < 0,05).

A prevalência de hipertensão, doença arterial coronariana e diabetes mellitus não diferiu significativamente entre sobreviventes e não sobreviventes (p > 0,05). No entanto, a mortalidade foi significativamente maior em pacientes com insuficiência cardíaca congestiva (p = 0,042) (Tabela 1). Em comparação aos sobreviventes, os não sobreviventes apresentaram níveis significativamente mais elevados de PCR, CAR, SUA/SCr e NPAR e níveis mais baixos de albumina sérica (todos com p < 0,001), além de níveis mais elevados de ferritina (p = 0,043) (Tabela 2).

**Tabela 2:** Associação dos reagentes de fase aguda e índices inflamatórios com a mortalidade.

Parâmetro	Estado de sobrevida	n	Média ± DP	Mediana	IIQ	Estatatística do teste	p
CRP (mg/L)	Sobrev.	100		6,0	3,0–13,8	858,0^*^	<0,001
	Não sobrev.	38		24,2	10,4–92,1		
Ferritina (ng/mL)	Sobrev.	100		270,0	137,5–601,0	1476,5^*^	0,043
	Não sobrev.	38		500,1	177,5–820,8		
Albumina (g/dL)	Sobrev.	100	3,52 ± 0,5			4,78^†^	<0,001
	Não sobrev.	38	2,89 ± 0,8				
CAR	Sobrev.	100		1,74	0,85–4,19	848,0^*^	<0,001
	Não sobrev.	38		8,32	3,31–38,45		
SUA/SCr	Sobrev.	100		0,66	0,55–0,77	940,0^*^	<0,001
	Não sobrev.	38		0,80	0,70–1,00		
NPAR	Sobrev.	100		17,31	15,6–20,0	1009,5^*^	<0,001
	Não sobrev.	38		21,41	18,49–33,22		
Parâmetro (categ.)	Corte	n	Mort. (%)			χ^2^	p
CAR	<3,1	8	10,4			25,67^¶^	<0,001
	≥3,1	30	49,2				
SUA/SCr	<0,716	10	13,9			14,05^¶^	<0,001
	≥0,716	28	42,4				
NPAR	<18,15	8	11,6			17,58^¶^	<0,001
	≥18,15	30	43,5				

Abreviaturas – Na seção categórica, n representa o número de pacientes que evoluíram a óbito, e Mort. (%) indica a mortalidade na respectiva categoria.DP: desvio-padrão; IIQ: intervalo interquartil; CRP: proteína C reativa; CAR: razão proteína C reativa/albumina; SUA/SCr: razão ácido úrico sérico/creatinina sérica; NPAR: razão percentual de neutrófilos/albumina; categ.: categórico. As variáveis categóricas foram definidas pelos valores de corte: CAR ≥ 3,1, SUA/SCr ≥ 0,716 e NPAR ≥ 18,15.

¶: teste do qui-quadrado (χ^2^);

†: teste t de *Student*;

*: teste de *Mann–Whitney* (U).

### Análise preditiva e fatores de risco para mortalidade

A análise por curvas ROC foi realizada para determinar os valores de corte ideais para SUA/SCr, CAR, NPAR e LDH (Figura 1). As taxas de mortalidade foram significativamente maiores em pacientes com CAR ≥ 3,1, SUA/SCr ≥ 0,716 e NPAR ≥ 18,15 (p < 0,001) (Tabela 2).

**Figura 1:** Determinação dos valores de corte ideais associados à mortalidade utilizando análise de curvas ROC (*receiver operating characteristic*).

Abreviaturas – A: Valor de corte da razão ácido úrico sérico/creatinina (SUA/SCr); B: Valor de corte da razão proteína C reativa/albumina (CAR); C: Valor de corte ideal da razão percentual de neutrófilos/albumina (NPAR); D: Valor de corte da desidrogenase láctica (LDH).

Na análise de regressão logística univariada, PCR, SUA/SCr, CAR e NPAR foram significativamente associados ao aumento da mortalidade. A ferritina apresentou associação limítrofe com a mortalidade (p = 0,050). Especificamente, SUA/SCr ≥ 0,716 foi associado a um aumento de 4,6 vezes no risco de mortalidade, CAR ≥ 3,1 a um aumento de 8,3 vezes, e NPAR ≥ 18,15 a um aumento de 5,9 vezes (Tabela 3).

**Tabela 3:** Associação entre as variáveis e a mortalidade: análise de regressão logística binária univariada.

Variável	B	E.P.	Wald	gl	Valor de p	Exp(B)	IC 95% para Exp(B)
Idade (anos)	0,029	0,014	4,466	1	**0,035**	1,029	1,002–1,057
Insuficiência cardíaca	0,862	0,430	4,027	1	**0,045**	2,369	1,020–5,500
PAS	-0,018	0,009	3,934	1	**0,047**	0,982	0,965–1,000
PAD	-0,021	0,015	1,955	1	0,162	0,979	0,951–1,008
HbA1c (%)	-0,226	0,159	2,004	1	0,157	0,798	0,584–1,091
Creatinina sérica	-0,419	0,098	18,381	1	**<0,001**	0,658	0,543–0,796
TFGe	0,088	0,044	4,025	1	**0,045**	1,092	1,002–1,190
Sódio (mmol/L)	0,084	0,063	1,745	1	0,187	1,087	0,960–1,231
LDH (U/L)	0,012	0,002	25,226	1	**<0,001**	1,012	1,007–1,016
Albumina	-1,744	0,401	18,919	1	**<0,001**	0,175	0,080–0,384
Leucócitos	0,000	0,000	5,896	1	**0,015**	1,000	1,000–1,000
Neutrófilos (%)	0,057	0,019	8,916	1	**0,003**	1,059	1,020–1,100
Linfócitos	0,000	0,000	2,202	1	0,138	1,000	0,999–1,000
PCR (mg/L)	0,029	0,007	14,863	1	**<0,001**	1,029	1,014–1,044
Ferritina	0,001	0,000	3,850	1	**0,050**	1,001	1,000–1,001
Acesso vascular (cateter)	1,290	0,431	8,962	1	**0,003**	3,634	1,561–8,456
Kt/Vureia	-3,891	1,149	11,466	1	**0,001**	0,020	0,002–0,194
SUA/SCr	2,845	0,914	9,688	1	**0,002**	17,204	2,868–103,198
SUA/SCr (categórica) ≥ 0,716	1,519	0,422	12,954	1	**<0,001**	4,568	1,997–10,448
CAR	0,072	0,021	11,829	1	**0,001**	1,074	1,031–1,119
CAR (categórica) ≥ 3,1	2,122	0,453	21,954	1	**<0,001**	8,347	3,436–20,277
NPAR	0,137	0,037	13,861	1	**<0,001**	1,147	1,067–1,233
NPAR (categórica) ≥ 18,15	1,769	0,448	15,619	1	**<0,001**	5,865	2,439–14,103

Abreviaturas – B: coeficiente de regressão; E.P.: erro-padrão; Wald: estatística de Wald; gl: graus de liberdade; Exp(B): *odds ratio*; IC 95%: intervalo de confiança de 95%; PAS: pressão arterial sistólica; PAD: pressão arterial diastólica; HbA1c: hemoglobina glicada; TFGe: taxa de filtração glomerular estimada; LDH: desidrogenase láctica; PCR: proteína C reativa; SUA/SCr: razão ácido úrico/creatinina; CAR: razão proteína C reativa/albumina; NPAR: razão percentual de neutrófilos/albumina. As categorias de referência foram: SUA/SCr < 0,716; CAR <3,1; NPAR < 18,15 e acesso vascular sem cateter.

Fatores potenciais de risco para mortalidade com valor de p < 0,25 nas análises univariadas foram posteriormente incluídos em um modelo de regressão logística multivariada. O modelo final apresentou bom ajuste, conforme evidenciado pelo teste de *Hosmer–Lemeshow* (χ^2^ = 1,67; p = 0,976). Nesse modelo multivariado, os preditores independentes de mortalidade foram identificados como LDH categórico ≥ 165,5 (OR = 21,81; p < 0,001), tipo de acesso vascular (cateter permanente [tunelizado]) (OR = 12,17; p = 0,007), nível de creatinina sérica (OR = 0,66; p = 0,013), razão SUA/SCr (OR = 4,84; p = 0,013), CAR categórico ≥ 3,1 (OR = 8,62; p = 0,005) e NPAR categórico ≥ 18,15 (OR = 3,81; p = 0,037) (Tabela 4).

**Tabela 4:** Associação entre as variáveis e a mortalidade com base na análise de regressão logística binária multivariada.

Variável	B	E.P.	Wald	gl	Valor de p	Exp(B)	IC 95% para Exp(B)
Idade (anos)	0,036	0,027	1,77	1	0,183	1,036	0,983–1,092
IC	-0,658	0,798	0,68	1	0,410	0,518	0,108–2,475
PAS	-0,046	0,028	2,72	1	0,099	0,955	0,904–1,009
PAD	0,068	0,050	1,89	1	0,169	1,071	0,971–1,180
HbA1c (%)	-0,394	0,349	1,28	1	0,258	0,674	0,340–1,336
Sódio (mmol/L)	0,153	0,117	1,71	1	0,191	1,165	0,927–1,465
LDH (categórico) ≥165,5	3,082	0,684	20,28	1	**<0,001**	21,808	5,702–83,417
Contagem de linfócitos	0,000	0,001	0,54	1	0,462	0,998	0,997–1,000
Ferritina	-0,001	0,001	0,63	1	0,429	0,999	0,998–1,001
Acesso vascular (cateter)	2,499	0,921	7,35	1	**0,007**	12,17	1,999–74,041
Creatinina sérica	-0,412	0,166	6,19	1	**0,013**	0,662	0,479–0,916
Kt/Vureia	-1,867	1,946	0,92	1	0,337	0,155	0,003–7,002
Contagem de leucócitos	0,000	0,000	3,20	1	0,074	1,000	1,000–1,001
SUA/SCr (contínuo)	1,578	0,636	6,15	1	**0,013**	4,843	1,392–16,853
CAR (categórico) ≥ 3,1	2,154	0,766	7,90	1	**0,005**	8,618	1,920–38,688
NPAR (categórico) ≥ 18,15	1,336	0,639	4,37	1	**0,037**	3,806	1,087–13,325

Abreviaturas – IC: insuficiência cardíaca; PAS: pressão arterial sistólica; PAD: pressão arterial diastólica; HbA1c: hemoglobina glicada; LDH: desidrogenase láctica; SUA/SCr: razão ácido úrico/creatinina; CAR: razão proteína C reativa/albumina; NPAR: razão percentual de neutrófilos/albumina.

### Análise de sobrevida

A análise de sobrevida de Kaplan–Meier revelou diferenças estatisticamente significativas nos tempos de sobrevida entre subgrupos de pacientes definidos pelos valores de corte de CAR (3,1), SUA/SCr (0,716) e NPAR (18,15), bem como pelo tipo de acesso vascular (SUA/SCr: log-rank χ^2^ = 16,96, p < 0,001; CAR: log-rank χ^2^ = 23,82, p < 0,001; NPAR: log-rank χ^2^ = 17,5, p < 0,001; acesso vascular: log-rank χ^2^ = 44,2, p < 0,001). As curvas de sobrevida correspondentes que ilustram esses resultados estão apresentadas na Figura 2.

**Figura 2:** Análise de sobrevida de *Kaplan–Meier* da mortalidade de acordo com razões inflamatórias e tipo de acesso vascular.

Abreviaturas – A: Curva de sobrevida de *Kaplan–Meier* de acordo com os valores categóricos da razão percentual de neutrófilos/albumina (NPAR); B: Curva de sobrevida de *Kaplan–Meier* com os valores categóricos da razão ácido úrico sérico/creatinina (SUA/SCr); C: Curva de sobrevida de *Kaplan–Meier* de acordo com os valores categóricos da razão proteína C reativa/albumina (CAR); D: Curva de sobrevida de *Kaplan–Meier* de acordo com o tipo de acesso vascular.

## DISCUSSÃO

Este estudo teve como foco a avaliação de indivíduos com doença renal em estágio terminal submetidos a um regime de hemodiálise três vezes por semana. Nossa análise demonstrou que a coorte de pacientes não sobreviventes apresentou níveis absolutos significativamente elevados de biomarcadores específicos, a saber, a razão ácido úrico sérico/creatinina sérica (SUA/SCr), a razão proteína C reativa/albumina (CAR) e a razão percentual de neutrófilos/albumina (NPAR). Além disso, após a aplicação dos valores de corte derivados da análise ROC, os modelos de regressão logística multivariada identificaram CAR e NPAR como fatores prognósticos independentes de mortalidade.

Os resultados deste estudo estão em concordância com a síndrome desnutrição–inflamação–aterosclerose (MIA), um conceito bem estabelecido em populações em diálise, no qual a inflamação sistêmica e o estado nutricional (refletido pelo IMC e pela albumina) atuam como determinantes críticos dos desfechos clínicos. Marcadores integrados como CAR e NPAR capturam efetivamente essa sinergia e funcionam como preditores independentes do risco de mortalidade.

A literatura existente indica que razões elevadas de SUA/SCr em pacientes em hemodiálise crônica estão associadas a um risco aumentado de morte cardiovascular^16^. Por exemplo, o estudo de Zhihui Ding et al.^17^ demonstrou uma forte associação entre a razão ácido úrico sérico/creatinina e a mortalidade por todas as causas em populações geriátricas em hemodiálise, destacando sua superior capacidade preditiva em comparação com o ácido úrico ou a creatinina isoladamente. Em concordância com essas observações, nossos dados revelaram razões de SUA/SCr marcadamente mais elevadas entre os indivíduos falecidos. Embora a análise de regressão logística multivariada tenha classificado a razão SUA/SCr como um preditor independente de mortalidade (OR = 4,8; p = 0,013), a avaliação categórica utilizando o valor de corte de 0,716 estabelecido pela análise ROC não demonstrou associação estatisticamente significativa com mortalidade no modelo multivariado (p = 0,380). A dicotomização de variáveis contínuas pode levar à perda de informação prognóstica, o que pode explicar a discrepância observada para SUA/SCr.

Estudos sugerem que níveis elevados de CAR estão significativamente associados tanto à mortalidade cardiovascular quanto à mortalidade por todas as causas, propondo o CAR como um indicador prognóstico independente em pacientes com doença renal crônica estágios 3–5^18^. Estudos adicionais caracterizam o CAR sérico como uma medida composta que reflete simultaneamente o estado nutricional e a carga inflamatória sistêmica, associando-o a maior risco de mortalidade em coortes de hemodiálise^10^. Nossos achados corroboram esses dados, demonstrando níveis significativamente mais elevados de CAR no grupo não sobrevivente (p < 0,001). Na análise multivariada, o CAR emergiu como um preditor independente de mortalidade. Especificamente, na análise categórica com valor de corte de 3,1 definido por ROC, indivíduos com CAR ≥ 3,1 apresentaram chances de mortalidade 8,6 vezes maiores (OR = 8,6; p = 0,005). Além disso, as curvas de *Kaplan–Meier* confirmaram uma redução significativa na sobrevida dos pacientes acima desse limiar (log-rank χ^2^ = 23,82; p < 0,001).

Estudos conduzidos por Changjing Shi et al.^19^ demonstraram que pacientes com razão NPAR acima do valor de corte de 16,96 apresentaram desfechos de sobrevida significativamente piores, identificando o NPAR como preditor independente de mortalidade. Em nossa análise, indivíduos com NPAR ≥ 18,15 apresentaram chances de mortalidade 3,8 vezes maiores (OR = 3,8; p = 0,037), sendo o NPAR confirmado como preditor independente no modelo multivariado. De forma consistente, a análise de *Kaplan–Meier* evidenciou redução significativa da sobrevida em pacientes com NPAR ≥ 18,15 (log-rank χ^2^ = 17,5; p < 0,001).

Naoki Kimata et al.^20^ demonstraram que reduções nos valores de Kt/Vureia estão significativamente associadas ao aumento da mortalidade, especialmente em populações femininas em hemodiálise, classificando essa redução como fator de risco independente. Em concordância com esses achados, nosso estudo demonstrou que os valores de Kt/V, como indicador de adequação dialítica, estavam significativamente mais baixos no grupo não sobrevivente (p < 0,001). No entanto, o modelo multivariado não evidenciou associação significativa entre Kt/V e mortalidade; portanto, o Kt/V não foi considerado um preditor independente de óbito (p = 0,337).

Observamos uma taxa de mortalidade de 38,2% em pacientes que utilizavam cateter para hemodiálise, em comparação com 14,5% entre aqueles com fístula arteriovenosa (AVF). O teste do qui-quadrado confirmou que essa diferença foi estatisticamente significativa (χ^2^ = 9,56; p = 0,002). Na regressão logística multivariada, o tipo de acesso vascular emergiu como fator de risco independente, sendo o uso de cateter associado a chances de mortalidade 12,2 vezes maiores (OR = 12,2; p = 0,007). Além disso, a análise de *Kaplan–Meier* demonstrou que pacientes com cateter apresentaram sobrevida significativamente menor em comparação ao grupo com AVF (log-rank χ^2^ = 44,2; p < 0,001). Esses resultados destacam o papel crítico do tipo de acesso vascular no prognóstico de pacientes em hemodiálise. Estudos prévios corroboram esses achados, demonstrando maior mortalidade associada ao uso de cateter em comparação com fístulas arteriovenosas^21,22^.

Níveis elevados de desidrogenase láctica (LDH) têm sido associados à mortalidade por todas as causas em estudos anteriores^23^. Por exemplo, Verena Gotta et al.^24^ observaram que níveis elevados de LDH em pacientes jovens e pediátricos em hemodiálise crônica podem atuar como um potente marcador de mortalidade, refletindo um perfil de risco complexo. Em concordância com esses achados, nosso estudo identificou níveis significativamente mais elevados de LDH nos pacientes não sobreviventes (p < 0,001). A análise de *Kaplan–Meier* demonstrou que indivíduos com LDH ≥ 165,5 apresentaram taxas de sobrevida significativamente menores (log-rank χ^2^ = 26,04; p < 0,001). Além disso, esses pacientes apresentaram chances de mortalidade 21,8 vezes maiores (OR = 21,808; p < 0,001), sendo o LDH confirmado como preditor independente no modelo multivariado.

### Limitações

Este estudo apresenta diversas limitações que devem ser consideradas na interpretação dos resultados. Primeiramente, seu delineamento retrospectivo e de centro único limita a generalização dos achados e impede inferências causais. Em segundo lugar, o tamanho relativamente pequeno da amostra e o número limitado de eventos de mortalidade podem aumentar o risco de sobreajuste nos modelos multivariados. Em terceiro lugar, os biomarcadores inflamatórios e metabólicos foram avaliados em um único momento, não sendo possível analisar sua variabilidade longitudinal, embora medições seriadas pudessem fornecer informações prognósticas mais robustas. Em quarto lugar, o desenho retrospectivo impediu o ajuste completo para potenciais fatores de confusão, incluindo uso de medicações (como estatinas, agentes estimuladores da eritropoiese ou terapias redutoras de ácido úrico), função renal residual e parâmetros de ultrafiltração. Além disso, apenas a mortalidade por todas as causas foi avaliada, impossibilitando a análise de desfechos específicos (como causas cardiovasculares ou infecciosas) e limitando a interpretação mecanística. Por fim, as análises de sobrevida foram restritas ao método de *Kaplan–Meier*, sem modelagem por riscos proporcionais de Cox, o que pode limitar a avaliação de efeitos dependentes do tempo. Portanto, é necessária a validação externa em coortes maiores, prospectivas e multicêntricas.

## CONCLUSÃO

Este estudo avaliou de forma abrangente biomarcadores associados à mortalidade em pacientes submetidos à hemodiálise e demonstrou que níveis elevados de SUA/SCr, da razão PCR/albumina (CAR), da razão percentual de neutrófilos/albumina (NPAR) e de LDH estão significativamente associados ao aumento da mortalidade. Pacientes com níveis de biomarcadores acima dos valores de corte definidos por análise ROC apresentaram taxas de sobrevida significativamente menores nas análises de *Kaplan–Meier*. Além disso, nos modelos de regressão logística multivariada, SUA/SCr contínuo, CAR, NPAR e LDH foram identificados como preditores independentes de mortalidade.

Adicionalmente, o uso de cateter venoso central foi associado a um risco significativamente maior de mortalidade em comparação com a fístula arteriovenosa. Esses achados ressaltam a importância de uma abordagem prognóstica integrada em pacientes em hemodiálise, considerando inflamação, estado nutricional, marcadores de lesão celular e tipo de acesso vascular. Biomarcadores amplamente disponíveis e de baixo custo podem auxiliar na estratificação de risco; entretanto, essa associação deve ser confirmada em estudos prospectivos multicêntricos de grande escala.

## Data Availability

Os conjuntos de dados analisados durante o presente estudo estão disponíveis com o autor correspondente, mediante solicitação razoável.

## MATERIAL SUPLEMENTAR

O seguinte material online está disponível para este artigo:

**Tabela S1** – Associação dos parâmetros laboratoriais com a mortalidade.
